# Evaluation of Methods for the Concentration and Extraction of Viruses from Sewage in the Context of Metagenomic Sequencing

**DOI:** 10.1371/journal.pone.0170199

**Published:** 2017-01-18

**Authors:** Mathis Hjort Hjelmsø, Maria Hellmér, Xavier Fernandez-Cassi, Natàlia Timoneda, Oksana Lukjancenko, Michael Seidel, Dennis Elsässer, Frank M. Aarestrup, Charlotta Löfström, Sílvia Bofill-Mas, Josep F. Abril, Rosina Girones, Anna Charlotte Schultz

**Affiliations:** 1 Research Group for Genomic Epidemiology, The National Food Institute, Technical University of Denmark, Kongens Lyngby, Denmark; 2 Division of Microbiology and Production, The National Food Institute, Technical University of Denmark, Søborg, Denmark; 3 Laboratory of Virus Contaminants of Water and Food, Department of Genetics, Microbiology, and Statistics, University of Barcelona, Barcelona, Catalonia, Spain; 4 Institute of Biomedicine of the University of Barcelona, University of Barcelona, Barcelona, Catalonia, Spain; 5 Institute of Hydrochemistry, Chair of Analytical Chemistry, Technical University of Munich, Munich, Germany; Sidra Medical and Research Center, QATAR

## Abstract

Viral sewage metagenomics is a novel field of study used for surveillance, epidemiological studies, and evaluation of waste water treatment efficiency. In raw sewage human waste is mixed with household, industrial and drainage water, and virus particles are, therefore, only found in low concentrations. This necessitates a step of sample concentration to allow for sensitive virus detection. Additionally, viruses harbor a large diversity of both surface and genome structures, which makes universal viral genomic extraction difficult. Current studies have tackled these challenges in many different ways employing a wide range of viral concentration and extraction procedures. However, there is limited knowledge of the efficacy and inherent biases associated with these methods in respect to viral sewage metagenomics, hampering the development of this field. By the use of next generation sequencing this study aimed to evaluate the efficiency of four commonly applied viral concentrations techniques (precipitation with polyethylene glycol, organic flocculation with skim milk, monolithic adsorption filtration and glass wool filtration) and extraction methods (Nucleospin RNA XS, QIAamp Viral RNA Mini Kit, NucliSENS^®^ miniMAG^®^, or PowerViral^®^ Environmental RNA/DNA Isolation Kit) to determine the viriome in a sewage sample. We found a significant influence of concentration and extraction protocols on the detected viriome. The viral richness was largest in samples extracted with QIAamp Viral RNA Mini Kit or PowerViral^®^ Environmental RNA/DNA Isolation Kit. Highest viral specificity were found in samples concentrated by precipitation with polyethylene glycol or extracted with Nucleospin RNA XS. Detection of viral pathogens depended on the method used. These results contribute to the understanding of method associated biases, within the field of viral sewage metagenomics, making evaluation of the current literature easier and helping with the design of future studies.

## Introduction

Within raw sewage, feces, urine and other biological fluids from thousands of humans are mixed together with food and household waste, industrial waste, and runoff water. Every individual, who is connected to the drainage system, contributes with his or hers own microbiota [1], including infecting pathogens [2]. This makes sewage an attractive matrix for epidemiological studies [3], microbial source tracking [4], and for controlling the efficacy of pathogen removal in wastewater treatment plants [5,6]. Sewage has been shown to harbor a diverse viral population including enteric, respiratory and oncogenic viruses [7]. The high viral diversity and the continuous mutation of viral species makes identification with traditional methods difficult and time consuming, therefore many studies have turned to Next Generation Sequencing (NGS) approaches instead [7–9]. Metagenomic sequencing of the virus associated nucleic acids is considered to be an unbiased approach enabling the detection of all known viral species, as well as the discovery of novel and emergent species [10]. Three main challenges exist for viral sewage metagenomics. First, only a small fraction of the total nucleic acids are of known viral origin, hence mechanical and enzymatic viral purification is often needed [9]. Second, the low abundance of viral particles in the samples requires the use of viral concentration methods prior to nucleic acid extraction [11] and is often combined with subsequent random DNA amplification [12]. Third, the nucleic acid extraction procedure has to cover the large variety in viral structures and genome types. To overcome these biases, different methods to concentrate viruses from water samples have been developed, including: polyethylene glycol precipitation (PEG) [8], FeCl_3_ precipitation [13], skimmed milk flocculation (SMF) [14], glass wool filtration (GW) [15] or monolithic adsorption filtration (MAF) [16]. The influence of concentration method on viral recovery has been evaluated on sea water [17], spiked tap water [15,18] and raw sewage [19], cautioning of method associated biases. To our knowledge, no major comparison studies using metagenomics have been performed with sewage water.

Biases caused by nucleic acid extraction kits have been well documented for both bacteria [20,21] and viruses [22,23]. In addition, contaminants have been found to be ubiquitous in some extraction kits [24] and laboratory reagents [25], potentially giving rise to false positive results [26,27]. A better understanding of specific method associated biases, in respect to viral wastewater metagenomics, would make evaluation of the current literature easier, and help guide future studies.

In this study we evaluated four previously published concentration methods, PEG, MAF, SMF, and GW, as well as four extraction kits, Nucleospin RNA XS (NUC), QIAamp Viral RNA Mini Kit (QIA), NucliSENS^®^ miniMAG^®^ (MIN), or PowerViral^®^ Environmental RNA/DNA Isolation Kit (POW), for wastewater viral metagenomics, in a full factorial design resulting in 16 combinations of procedures. Aspects studied included viral community composition, viral selectiveness, viral richness, viral pathogen detection, and viral contaminants. Extracted nucleotides were amplified with PCR and sequenced using the Illumina MiSeq platform.

## Materials and Methods

### Sample collection, spiking and pooling

In July 2015 raw sewage (130 L) was collected at the waste water treatment plant BIOFOS Lynetten in Copenhagen, Denmark, receiving waste water from about 550,000 inhabitants. Approval was granted from BIOFOS Lynettefællesskabet A/S before sampling. The sewage was mixed thoroughly in a single container and spiked to a concentration of 1.74×10^8^ RT-PCR units/L of murine norovirus (MNV) (kindly provided by Dr Virgin, Washington University School of Medicine, USA), and 2.13×10^9^ genome copies/L of human adenovirus 35 (HAdV). The sample was mixed for 5 min before aliquoted and stored at—20°C until further processing.

### Concentration methods

Four different methods were used to concentrate virions from the sewage samples: protein precipitation with PEG, organic flocculation with SMF and filtration with positively charged filters, MAF, or GW. All concentration methods were done in triplicate together with a negative control using sterile molecular grade water (VWR—Bie & Berntsen, Søborg, Denmark).

### PEG

The PEG protocol was based on the procedure as previously described [8]. Initially, 25 mL of glycin buffer (0.05 M glycine, 3% beef extract, pH 9.6) was added to 200 mL of sewage and mixed, to detach virions bound to organic material. The sample was then centrifuged at 8,000×g for 30 min, and the collected supernatant was filtered through a 0.45 μm polyethersulfone (PES) membrane (Jet Biofil, Guangzhou, China) to remove bacterial and eukaryotic cells. Viruses were precipitated from the supernatant by incubation with PEG 8000 (80 g/L) and NaCl (17.5 g/L) during agitation (100 rpm) overnight at 4°C, followed by centrifugation for 90 min at 13,000×g. The resulting viral-containing pellet was eluted in 1 mL phosphate buffer saline (PBS) and stored at -80°C until further processing.

### MAF

The principle of the MAF adsorption/elution method was based on the procedure as previously described [18]. Monolithic discs, diameter 3.86 cm and height 1.0 cm, were synthesized by polymerization of polyglycerol-3-glycidyl ether (Ipox chemicals, Laupheim, Germany). An 80:20 mixture of toluene and tert-buthyl methyl ether was used as porogen to create monoliths with a pore size of ca. 20 μm. After synthesis, functionalization was performed by recirculating 10% diethylamine in 50% ethanol at 60°C through the monolithic disks for 3 h to create positively charged diethylaminoethyl groups on the pore surface. Afterwards the monoliths were rinsed with ultrapure water and stored at 4°C until further use. One liter of raw sewage was filtrated through a MAF disc (Microarray and Bioseparation Group of the Institute of Hydrochemistry, Technical University of Munich, Germany) assembled as previously described [28]. Viruses were eluted from the filter by soaking 2×2 min in a total of 20 mL high salt buffer (1.5 M NaCl, 0.05 M HEPES (4-(2-hydroxyethyl)-1-piperazineethanesulfonic acid) buffer, pH 7). The eluate was further concentrated to 3 mL by 100 kDa Amicon ultra centrifugation filters (Merck Millipore, Cork, Ireland) according to the manufacturer’s instructions. The viral concentrate was stored at -80°C until further processing.

### SMF

Organic flocculation with skimmed milk was based on the procedure as previously described [7]. In brief, 100 mL pre flocculated skimmed milk solution (1% (w/v) skimmed milk powder (Difco, Detroit, MI, USA), 3.2% (w/w) sea salts (Sigma Aldrich Chemie GMBH, Steinheim, Germany)) at pH 3.5 was added to 10 L of acidified (using HCl to pH 3.5) raw sewage and mixed for 8 h. Flocculants were allowed to sediment for 8 h, and centrifuged at 8,000×g for 40 min. The pelleted viral concentrate was suspended in 15 mL phosphate buffer (1:2 (v/v) mixture of 0.2 M Na_2_HPO_4_ and 0.2 M NaH_2_PO_4_). The phosphate suspension was eluted in 30 mL 0.25 M glycine buffer (pH 9.5) with slow agitation for 45 min at 4°C. Suspended solids were separated by centrifugation at 8,000×g for 40 min at 4°C. The sample was neutralized to pH 7 by adding 1 M HCl. Virions present in the supernatant were concentrated by ultracentrifugation at 90,000×g for 90 min (Sorvall Discovery 90SE) at 4°C and suspended in 2 mL PBS. The viral concentrate was stored at -80°C until further processing.

### GW

The glass wool filters were prepared as previously described [29]. Sodocalcic glass wool (15 g) (Ouest Isol, Alizay, France) was packed into a PVC tube with the density of 0.11 g/cm^3^ and pretreated with the following solutions, 100 mL NaOH (1 M) for 15 min, 1 L sterile distilled water, 100 mL HCl (1 M) for 15 min, and 1 L sterile distilled water. Samples of raw sewage (4 L) were filtered through the glass wool column. Viruses were eluted by incubating 100 mL elution buffer (3% beef extract, 0.5 M glycine, pH 9.5) for 15 min. Secondary concentration was done by PEG precipitation (as above) to a final volume of 1 mL.

### DNase/RNase treatment + chloroform-butanol treatment

All viral concentrates were treated with OmniCleave endonuclease (Epicentre, Wisconsin, USA) to remove extracellular DNA/RNA as previously described [30]. Samples were further purified by extraction using a 1:1 mixture of chloroform-butanol [31] to remove nucleases and inhibitors.

### Extraction methods

Nucleic acids were extracted from 200 μL-portions of the respective viral concentrate using four different extraction kits; NUC (Macherey-Nagel, Düren, Germany), QIA (Qiagen, Valencia CA, USA), MIN (BioMerieux, Herlev, Denmark) or POW (MO BIO, Carlsbad, CA, USA). In all cases, extractions were carried out according to manufacturer’s instructions.

### qPCR analysis of spiked viruses

Detection of HAdV and MNV was performed on extracted nucleic acids (undiluted and 10-fold diluted) in a 96-well plate format of ABI Step One (Applied Biosystems, Naerum, Denmark). MNV RNA was detected by quantitative reverse transcriptase polymerase reaction (qRT-PCR) using the RNA UltraSense one-step qRT-PCR system (Invitrogen, Taastrup, Denmark) and previously described primers and probes [32]. Amplification was performed in a 25 μL reaction mixture containing 5 μL extracted nucleic acids and 20 μL qRT-PCR reaction mixture with 500 nM forward primer, 900 nM reverse primer, 250 nM probe, 1 × UltraSense reaction mix, 1 × ROX reference dye and 1 × UltraSense enzyme mix under the following reaction conditions, 55°C for 1 min and 95°C for 5 min followed by 45 cycles of 95°C for 15 s, 60°C for 1 min, and 65°C for 1 min. HAdV DNA was detected by qPCR using TaqMan Universal Master Mix (Applied Biosystems, Naerum, Denmark), and previously described primers and probe [33]. Amplification was performed in a total of 25 μL reaction mixture containing 5 μL extracted nucleic acids and 20 μL qPCR reaction mixture containing 1 × TaqMan Universal Master Mix, primer and probe concentrations and qPCR running conditions are described in [33]. Quantification was performed using standard curves generated from 10-fold dilution series, of extracted RNA of cell propagated MNV or of ds HAdV DNA segments, artificially constructed by gBlocks^®^ Gene Fragments (Integrated DNA Technologies, Leuven, Belgium).

### Reverse transcriptase, library preparation and, sequencing

To prepare extracted RNA and DNA for sequencing, each viral extract was subjected to reverse transcriptase and PCR amplified, as previously described [34]. Briefly, first strand cDNA synthesis were performed using the SuperScript^®^ III First-Strand Synthesis SuperMix (Invitrogen, Carlsbad, California) and 1 μL Primer A (50 μM) (5’-GTTTCCCAGTCACGATCNNNNNNNNN-3’) according to the manufacturer’s instructions. Second strand DNA synthesis were performed using Klenow Fragment exo-polymerase (Thermo Fisher Scientific, Waltham, MA, USA) as previously described [30]. Double stranded DNA products were PCR amplified using AmpliTaq Gold (Qiagen, Valencia CA, USA) as per manufacturer’s instruction using 0.8 μM Primer B (5′- GTTTCCCAGTCACGATC -3′) and the following conditions, 10 min at 95°C, 25 cycles of amplification (94°C for 30 s, 40°C for 30 s, 50°C for 30 s and 72°C for 1 min), and 1 cycle of elongation (72°C for 10 min). PCR products were purified using the DNA Clean & Concentrator^™^-5 (Zymo Research, Irvine CA, USA). NGS library preparation was performed using the Nextera XT DNA Library Preparation kit (Illumina, Eidenhoven, The Netherlands) according to the manufacturer’s instructions. The 64 samples were sequenced on three Illumina MiSeq runs with an average output of 1.4 × 10^6^ 250 bp paired-end reads per sample (S1 Table).

### Bioinformatic analyses

The distribution of viral species was determined using MGmapper software version 2.2 (https://cge.cbs.dtu.dk/services/MGmapper/) [31]. The MGmapper tool follows three main steps: quality assessment of the raw reads, mapping of reads to the reference databases, and post-processing of mapping results. Quality assessment was done using cutadapt [35] which performs common adapter removal, trimming of the low-quality ends from reads with a minimum Phred quality score of 20, and later discards reads that are shorter than 40 bp. Later, already trimmed pair-end reads were aligned to a pre-defined set of reference sequence databases using bwa mem [36] ver. 0.7.7-r441 with default settings. In this study, reads were mapped against three viral reference databases (S2 Table): whole genomes virus sequences (Virus) and viral sequences extracted from nt database (Virus_nt), obtained from Genbank (http://www.ncbi.nlm.nih.gov/genbank/), as well as Vipr database (http://www.ncbi.nlm.nih.gov/pmc/articles/PMC3245011). Samtools [37] were used to remove singletons and filter reads where neither a read nor its mate is mapped. Reads were mapped in best-mode, meaning that mapping was performed against all databases, simultaneously, and later for each read pair the best hit among all alignments is chosen. A pair of reads is considered as a hit only if the sum of the alignment scores (SAS) is higher than any SAS values from other database hits. If a pair of reads has identical SAS values when mapping to several databases, the only one pair, associated with the database that was specified first in the list of reference databases, is kept. In the last, post-processing step, alignments are filtered based on matches/mis-matches threshold. In this analysis, 70% matches/mis-matches threshold needed to be satisfied in order the hit to be considered significant. The metagenomic sequences are available from the European Nucleotide Archive (ENA) at the European Bioinformatics Institute (EBI) under accession number PRJEB15242.

### Statistics and plots

Viral richness was estimated using the program CatchAll [38] and the non-parametric Chao1 richness index, as a measure of number of viral species in a sample. All statistics were done in R [39], using two-way analysis of variance (ANOVA) test for determining the overall significance of concentration or extraction method on the studied factors (viral richness, etc.). Subsequently, pairwise t-tests with “Holm-Bonferroni” [40] p-value adjustments were applied to determine significant pairwise effects between individual concentration or extraction methods. Principal component analysis (PCA) were performed using prcomp and plotted with ggbiplot [41]. Heatmaps were created using pheatmap [42]. Linear regression between reads per million (RPM) and genome copies per liter were done in Excel on log transformed data.

## Results

In this study different virus concentration and nucleic acid extraction methods were evaluated for metagenomic analysis of sewage samples. Sequencing results showed that the majority of the mapped reads (>80%) were of viral origin (S1 Fig). However, between 60 and 90% of the total reads were unmapped. The three main viral families detected were Adenoviridae (human viruses) including the spiked HAdV 35, Virgaviridae (plant viruses), and Siphoviridae (bacteriophages). The sequencing data were analyzed further to determine the viral community composition, viral specificity, viral richness, and detection of pathogenic species.

### Viral community composition

**Fig 1:**
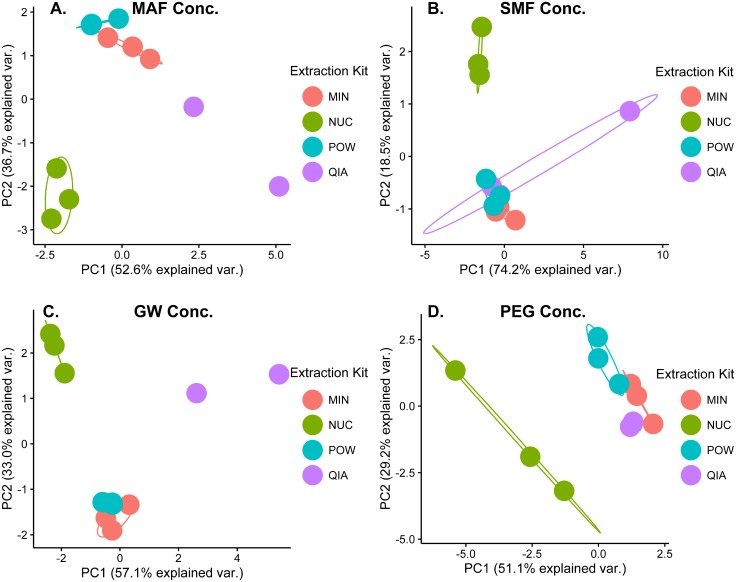
The influence of extraction method on the viral community composition. PCA plots made by using the relative abundances of the nine most abundant viral families. Separate PCAs were done for (A) samples concentrated with MAF, (B) SMF, (C) GW, and (D) PEG. Sample replicates were individually plotted and grouped according to the extraction method. In cases where only two samples were present, no ellipse representing the cluster was drawn.

**Fig 2:**
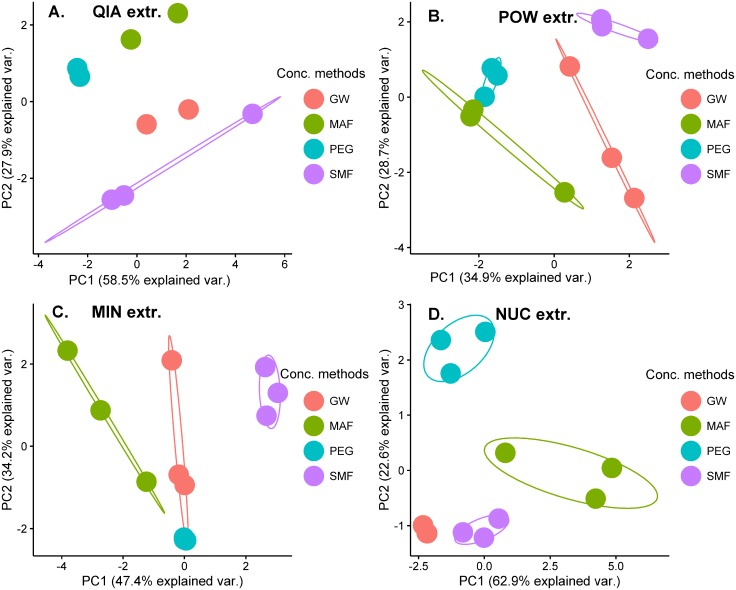
The influence of concentration method on the viral community composition. PCA plots made by using the relative abundances of the nine most abundant viral families. Separate PCAs were done for (A) samples extracted with QIA, (B) POW, (C) MIN, and (D) NUC. Sample replicates were individually plotted and grouped according to the concentration method. In cases where only two samples were present, no ellipse representing the cluster was drawn.

To compare the viral community composition resulting from the individual concentration and extraction methods, a series of PCAs were made using the relative abundances from the nine most abundant viral families, accounting for more than 99% of the mapped viral sequences. The effect of extraction (Fig 1) and concentration (Fig 2) were plotted independently for easier visualization. Samples plotted close together have similar viral community compositions, whereas samples far away from each other are less alike. The negative controls clustered together far away from the samples in initial PCA plots (data not shown). To allow for better visualization of the effect of concentration and extraction on the sewage samples, they were not included in Figs 1 and 2.

Sample replicates extracted with NUC clustered away from the samples extracted with the other methods, when concentrated with PEG (Fig 1D). This was also true for the concentrates from MAF (Fig 1A), SMF (Fig 1B), and GW (Fig 1C), suggesting that the viral community composition of the NUC extractions differed from the other tested extraction methods. The samples extracted with POW and MIN clustered together, suggesting similar viral community compositions (Fig 1A–1D). The samples extracted with QIA sometimes clustered separately (Fig 1A and 1C) and sometimes together with the samples extracted with POW and MIN (Fig 1B and 1D). The four concentration methods formed separate non-overlapping clusters regardless of extraction kit used (Fig 2A–2D), although some variation between replicates were observed.

### Viral specificity

The proportion of reads mapping to viruses ranged between 3.4% and 49.4%. Both the concentration and the extraction methods had a statistical significant effect on the viral specificity (two-way ANOVA, p < 0.001). However, a significant interacting effect (two-way ANOVA, p < 0.001) indicated that the effect on viral specificity by the extraction method was affected by the type of concentration method, and vice versa.

**Fig 3:**
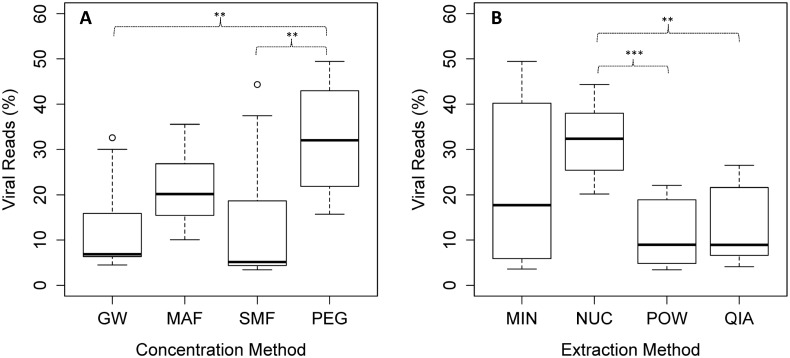
Viral selectivity measured in percentage of reads. (A) Viral selectivity for the tested concentration methods (B) and extraction methods. Each boxplot was made from 12 individual samples (including the four extraction/concentration methods with three replicates each). The bar, box, whiskers and circles represents median, inter-quartile range, inter-quartile range times 1.5, and outliers, respectively. Asterisks represent significance level of a pairwise t-test with “Holm-Bonferroni” adjusted p-values. ** = p < 0.01, *** = p < 0.001.

The PEG concentration method had a significant larger mean proportion of viral reads compared to the SMF and GW methods (pairwise t-test, p < 0.01) (Fig 3A). For the extraction methods, NUC had a significant larger mean proportion of viral reads compared to POW and QIA (pairwise t-test, p < 0.01) (Fig 3B). However, there were some interacting effects, with MIN scoring higher than NUC when used in combination with PEG and GW, implying that the MIN method depends heavily on the performance of the concentration method.

### Viral richness

**Fig 4:**
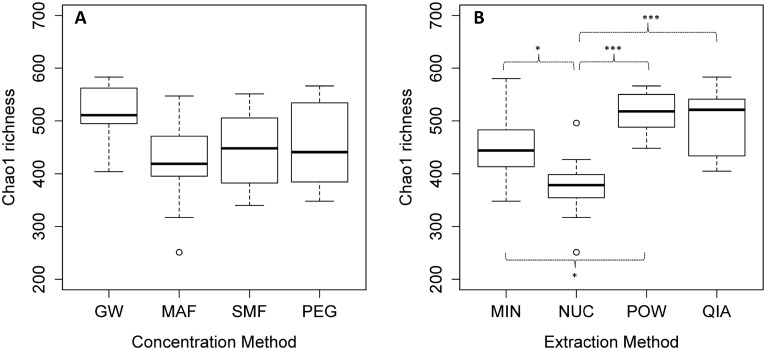
Viral species richness. (A) Viral Chao 1 species richness of the tested concentration methods, and (B) extraction methods. Each boxplot was made from 12 individual samples (including the four extraction/concentration methods with three replicates each). The bar, box, whiskers and circles represents median, inter-quartile range, inter-quartile range times 1.5, and outliers, respectively. Asterisks represent significance level of a pairwise t-test with “Holm-Bonferroni” adjusted p-values. * = p < 0.05, ** = p < 0.01, *** = p < 0.001.

Both concentration and extraction methods had an effect on the viral richness. However, none of the concentration methods were statistically different from each other (pair-wise t-test) (Fig 4A). For the extraction methods, NUC had a significantly lower Chao1 richness than the other methods (Fig 4B). POW and QIA had the highest mean richness estimates of 516 and 495, respectively.

### Detection of pathogenic species

**Fig 5:**
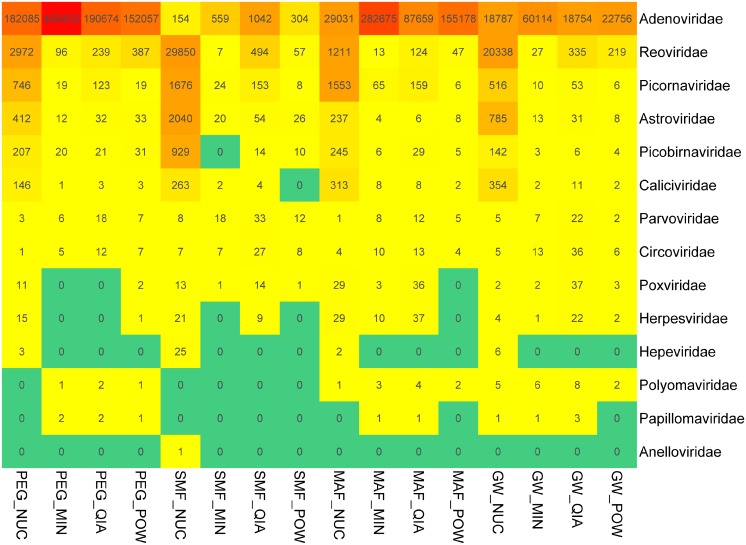
Detection of pathogenic viral families. Heatmap of the relative abundance of 14 human pathogenic viral families, detected by the 16 different concentration/extraction combinations. The numbers within each cell represents reads per million. The colours range from green = no detection, to red = high relative abundance.

Fourteen viral families with suspected human pathogens were detected (Fig 5). The most prevalent was Adenoviridae including the spiked HAdV. The highest read count for the viral RNA families, Reoviridae, Picornaviridae, Astroviridae, Caliciviridae and Picorbinaviridae, was obtained in samples extracted with NUC. The spiked HAdV was detected at the highest abundance when extracted with MIN. The effect of the concentration methods was not as pronounced as for the extraction kits. The highest read count of the DNA virus family, Adenoviridae, was found in samples concentrated with MAF and PEG. In general, SMF had a lower performance compared with the other methods when testing for Adenoviruses such as the spiked HAdV. However, the combination of SMF and NUC had the highest read count for most of the RNA viruses.

The spiked MNV were only detected by metagenomics in 57% of the samples, and at low read counts, from 2 to 194 reads. The combinations that could detect most MNV were MAF and GW extracted with NUC as well as MAF extracted with QIA and MIN.

### qPCR analysis of spiked viruses

The detected concentrations of HAdV and MNV varied widely between the different method combinations (S2 and S3 Figs) with mean values ranging between 650 and 8.2 × 10^7^ genome copies/L for HAdV, and 1.8×10^2^ and 3.9 × 10^5^ RT-PCR units/L for MNV. Choice of extraction method did not influence HAdV or MNV recovery. However, concentration methods had a significant impact (pairwise t-test, p < 0.05). The highest recovery of HAdV and MNV was obtained with PEG followed by MAF, GW, and SMF.

### Inhibition

To investigate the possibility of PCR inhibition, extracts of nucleic acids (undiluted and 10-fold diluted) from all samples were analyzed for the two spiked viruses, MNV and HAdV, with qPCR (S3 Table). The lowest inhibition of MNV and HAdV were observed in samples concentrated with PEG or extracted with MIN. Strongest inhibition was observed in samples concentrated with SMF for both MNV and HAdV. In addition samples extracted with QIA showed strong inhibition of HAdV detection.

### Correlation between qPCR quantification and reads per million (RPM)

To investigate the correlation between viral concentrations and RPM, qPCR data was compared with read counts from the two spiked viruses, HAdV and MNV. There was a strong correlation between RPM and qPCR enumeration for HAdV (R^2^ = 0.82). However, no relationship was observed for MNV (R^2^ = 0.07).

### Contamination

To detect method dependent contamination, a negative control was included, using sterile molecular grade water, for each of the 16 method combinations. Negative controls generally had a low total read count, a low percentage of viral reads (0.05–3.4%), and a high abundance of reads with human, bacterial, fungal and parasitic origin (S1 Fig). Several viral species were found in the negative controls with much higher RPM values than in the corresponding samples, suggesting that they originated from the corresponding kits or reagents. Reads mapping to pandora viruses, tupaiid herpes viruses, and *Citrobacter* phages were contaminants in all procedures except the ones using QIA extractions. However several mardi viruses were found exclusively in the QIA negative controls.

## Discussion

In the presented study we evaluated the influence of four commonly applied concentration and extraction methods on viral metagenome analysis.

The viral community composition was heavily biased by the type of concentration procedure, which dramatically skewed the relative abundances (Fig 2). Choice of extraction kit did not influence the viral community composition to the same degree (Fig 1). However, the results from the NUC extraction kit were remarkably different from samples extracted with the three other kits. The NUC kit includes an “on column DNase step” after viral capsid disruption, which selects for RNA viruses and could explain the separate clustering in the PCA plots (Fig 1). Based on the results from this study it seems inadvisable to compare results, in relation to viral community composition, between studies using different concentration methods and to some degree also extraction methods.

A high species richness have been linked to several ecosystem functions [43], and is often included as a factor in ecological studies. In this study we included the measure to discern if some methods were better at catching the entire spectrum of viral species. Our results show that the choice of extraction method is of more importance than the choice of concentration method with regard to viral richness. However, samples concentrated with GW had a slightly higher richness compared to the other concentration methods (Fig 4A). The low mean richness of the samples extracted with NUC can probably be explained by the DNase step, degrading the genomes of DNA viruses and the species rich bacteriophages [44].

Viral specificity, or how large a fraction of the sequencing reads is of viral origin, is important for sensitivity reasons, increasing the chance to detect rare or less abundant species. A high viral specificity also has financial implications, causing large savings on both sequencing, and for subsequent CPU hours used in the bioinformatics analyses. In this study, the PEG protocol was the best concentration method, in respect to viral specificity (Fig 3A). This might be explained by the initial filtration step, not part of the other evaluated protocols. Pre-filtration might have improved the viral specificity in the other concentration methods, although clotting might become a problem due to the increased volumes processed with these methods. The NUC had a consistent high viral specificity (three times that of POW and QIA), probably due to the effective removal of DNA from other organisms, and contaminants, during the DNase step. Overall, there was a 10-fold difference in viral specificity between the lowest and the highest method combination, highlighting the potential savings associated with choice of method. We observed a generally high viral specificity in this study compared to previous studies [45]. This might be due to the addition of the spiked HAdV, inflating the amount of virus in the sewage matrix, but should not have any influence on the method comparisons.

Sewage metagenomics is often used to detect human viral pathogens [8] including the important enteric RNA viruses as norovirus [46], rotavirus [47] and Hepatitis A and E virus [48] that has a big impact on public health [49]. These RNA viral families were best detected when using the NUC extraction kit compared to the other tested extraction kits, especially in combination with the concentration method SMF. However, if looking at DNA viruses exclusively, the MIN extraction combined with PEG, MAF, or GW may be preferable, since it produced the highest read counts for the spiked Adenoviridae. Low detection of Adenoviruses using SMF concentration has previously been described [7], and were also observed in this study. In addition, SMF failed to detect the low numbers of reads of polyomaviruses and papillomaviruses observed by the other methods.

The larger initial sample volume, and associated organic material and inhibitors, for SMF (10 L) compared with the other methods (4, 1 and 0.2 L for GW, MAF, and PEG, respectively), could be an explanation for the low recovery of the spiked viruses. Inhibitors can affect PCR amplification, quality of the prepared library, and subsequent virus detection. This theory was further supported by the qPCR results were extracts obtained from SMF had a high level of inhibition. Extraction with QIA has previously been shown to impair detection of HAdV in samples with high levels of organic matter [23]. This was also the case in our study, where extraction with QIA inhibited HAdV detection in all cases except when combined with PEG concentration which both had the lowest starting volume (0.2 L) and an additional filtration step.

Sampling volume is an important factor in viral metagenomics, enhancing the sensitivity and increasing the chances of detecting rare viruses. However, in this study, we did not find a positive relation between methods with high sampling volumes and increased sensitivity. This could be due to an increase in inhibitors or other aspects of the employed concentration methods, although this question was not within the scope of this study. Further studies are needed to investigate the influence of sample volumes and viral metagenomics.

In this study, the bioinformatic analyses were done using alignment of single reads to three virus databases, using the program MGmapper. The choice of bioinformatics pipeline can affect results [50] but any biases of our particular approach should be the same on all samples and should therefore not affect the conclusions of this study.

Low levels of MNV were detected in the metagenomics analysis compared to the amounts used for spiking. However, the reasonable high values that could be detected using qPCR, indicated that the initial extraction was successful. Noroviruses have previously been documented to be difficult to detect using metagenomics [51,52] possibly because of the small genome, robust nucleocapsid, or inhibitory RNA secondary structures [53]. Virus species specific extraction efficiency biases are well documented in viral metagenomics [54] and should always be considered when interpreting the results. Quantitative conclusions from viral metagenomics are not possible for all viral species, illustrated by the good correlation between RPM and qPCR data found for HAdV where no correlation was found for MNV.

Several viruses were detected in higher amounts in the negative controls than in the corresponding samples, strongly suggesting them to be procedure contaminants. Contaminating DNA is a huge challenge for low input metagenomics [24], and contaminating viral nucleotides have previously been detected in polymerases [25], spin columns [27] and DNases [54]. The specific origin of the contaminating viruses in our study was not clear although some avian herpesviruses were only linked to the QIA extracts. The ubiquitous presence of contaminating viruses stress the importance of including negative controls in future viral metagenomics studies, as well as adding measures to reduce the problem [55,56].

When evaluating the efficiency of the tested methods, clear differences were observed. No single method was superior to the others in all of the tested parameters. However, some trends were observed for the concentration methods as PEG scored higher in viral specificity and SMF inhibited detection of both spiked viruses. In the evaluation of the tested extraction methods the NUC kit stood out in regard to viral specificity and RNA virus detection. Nevertheless, if the focus is only on DNA viruses, for example phage studies, NUC might not be the best option since it scored low in viral richness which could result in loss of rare species. Practical aspects of the concentration and extraction methods were not within the scope of this paper, but may also influence the choice of method (S4 and S5 Tables).

In conclusion, we found a significant influence of concentration and extraction protocols on viral richness, viral specificity, viral pathogen detection, and viral community composition for metagenomic analyses of sewage. This is of major importance when interpreting results from the literature and conducting meta-studies. The use of data base resources, such as the European nucleotide archive (ENA) and short read archive (SRA) are also severely hampered by this fact since extraction kit, volume sample, and concentration procedure are not usually included in the metadata of published viromes. We suggest that such metadata will be included in the future, to allow researchers to select and compare studies conducted with similar methodologies.

## Supporting Information

S1 Fig Distribution of reads on kingdom level of the 16 method combinations and their associated negative controls. Samples were processed in triplicate, and the data shown is the average. _S = sample, _C = Negative extraction control. Databases used are listed in S1 Table.
(PDF)

S2 Fig HAdV concentration measured by qPCR. (A) HAdV concentration in extracts obtained by using four different concentration methods and (B) extraction methods. The bar, box, whiskers and circles represents median, inter-quartile range, inter-quartile range times 1.5, and outliers, respectively. Asterisks represent significance level of a pairwise t-test with “Holm-Bonferroni” adjusted p-values. ** = p <0.01, *** = p < 0.001.
(PDF)

S3 Fig MNV concentrations measured by qPCR. (A) MNV concentration in extracts obtained by using four different concentration methods and (B) extraction methods. The bar, box and whiskers represents the median, the inter-quartile range, and the inter-quartile range times 1.5, respectively. Asterisks represent significance level of a pairwise t-test with “Holm-Bonferroni” adjusted p-values. * = p < 0.05, ** = p < 0.01, *** = p < 0.001.
(PDF)

S4 Fig Abundance of all detected viral families. Heatmap showing the abundance of all detected viral families, measured in reads per million, in each biological replica for the different method combinations as well as the controls. _S = sample, _C = Negative control.
(PDF)

S1 Table Sequence information. Number of raw reads, reads after quality assessment, and reads not mapping to PhiX, and thus usable for subsequent analysis. _S = sample, _C = Negative control.
(PDF)

S2 Table Overview of reference sequence databases and associated download information. Reference sequence information can be obtained from the URL’s shown in ‘Download information’.
(PDF)

S3 Table qPCR inhibition of MNV and HAdV. Inhibition of the 16 combinations of concentration and extraction methods. Inhibition was measured using qPCR of undiluted (1:1) and tenfold diluted (1:10) DNA/RNA extracts. The values in the tables represents Δct between the undiluted and 10 fold diluted samples. A Δct = -3.3 represent a perfect 10 fold dilution. Samples marked in red represents undiluted extracts that could not be quantifiable, these samples are regarded as the most inhibited.
(PDF)

S4 Table Specifications of the four concentration methods applied in this study. (PDF)

S5 Table Properties of the four nucleic acid extraction kits applied in this study. (PDF)
