# Supplementary material for: Evaluation of Methods for the Concentration and Extraction of Viruses from Sewage in the Context of Metagenomic Sequencing
Source: PLoS One. 2017 Jan 18;12(1):e0170199. doi: 10.1371/journal.pone.0170199 (PMC5242460; doi:10.1371/journal.pone.0170199)
Supplement: S1 Fig — Samples were processed in triplicate, and the data shown is the average. _S = sample, _C = Negative extraction control. Databases used are listed in S1 Table. (PDF) [file pone.0170199.s001.pdf]

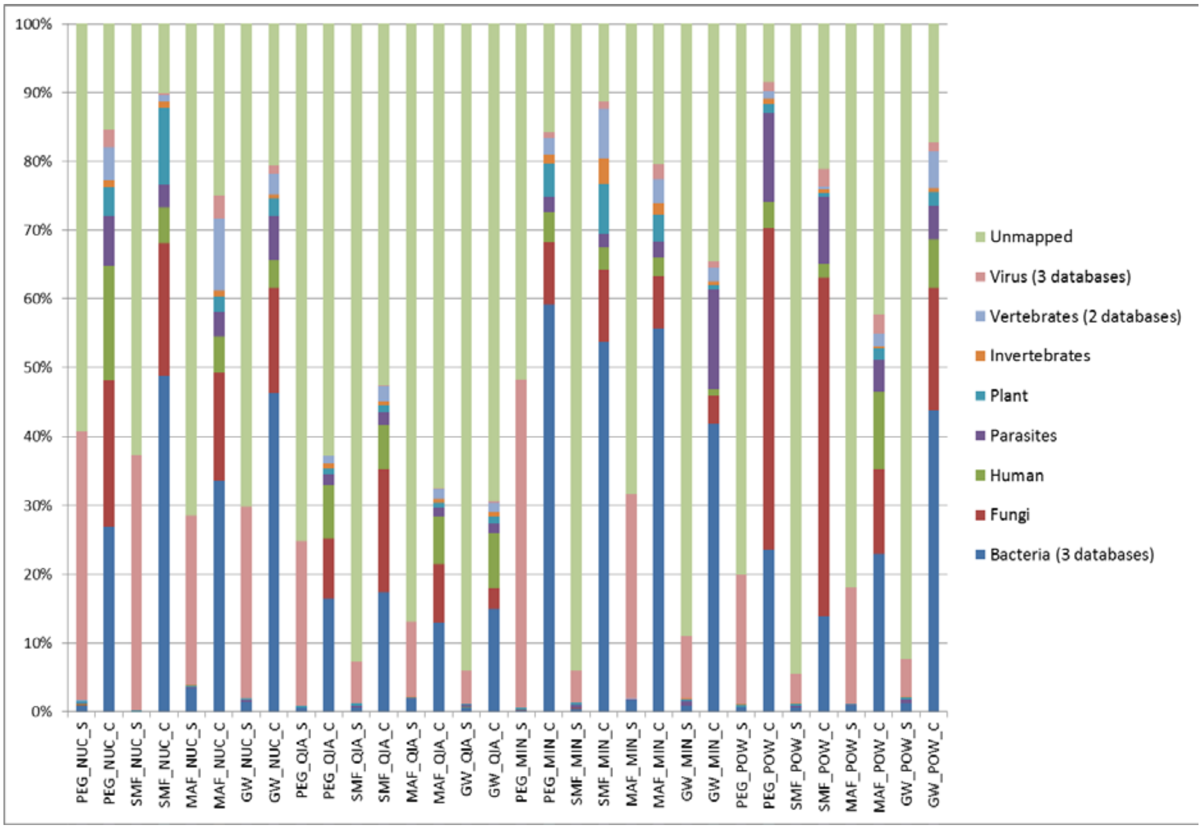

S1 Fig. Distribution of reads on kingdom level of the 16 method combinations and their associated negative controls. Samples were processed in triplicate, and the data shown is the average. _S = sample, _C = Negative extraction control. Databases used are listed in S1 Table.
