# Supplementary material for: Evaluation of Methods for the Concentration and Extraction of Viruses from Sewage in the Context of Metagenomic Sequencing
Source: PLoS One. 2017 Jan 18;12(1):e0170199. doi: 10.1371/journal.pone.0170199 (PMC5242460; doi:10.1371/journal.pone.0170199)
Supplement: S2 Fig — (A) HAdV concentration in extracts obtained by using four different concentration methods and (B) extraction methods. The bar, box, whiskers and circles represents median, inter-quartile range, inter-quartile range times 1.5, and outliers, respectively. Asterisks represent significance level of a pairwise t-test with “Holm-Bonferroni” adjusted p-values. ** = p <0.01, *** = p < 0.001. (PDF) [file pone.0170199.s002.pdf]

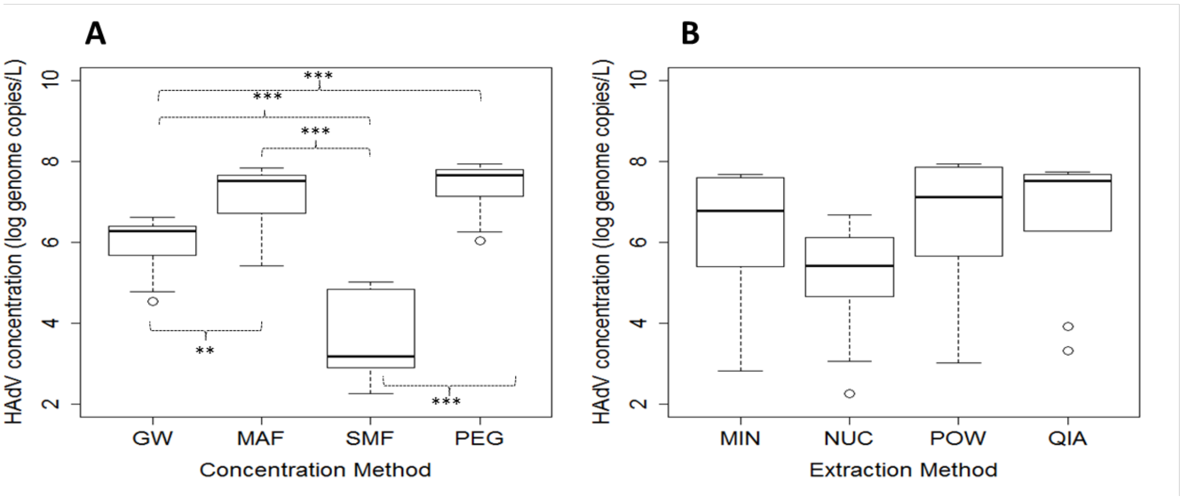

S2 Fig. **HAdV concentration measured by qPCR.** (A) HAdV concentration in extracts obtained by using four different concentration methods and (B) extraction methods. The bar, box, whiskers and circles represents median, inter-quartile range, inter-quartile range times 1.5, and outliers, respectively. Asterisks represent significance level of a pairwise t-test with "Holm-Bonferroni" adjusted p-values. ** = $p < 0.01$, *** = $p < 0.001$.
