# Supplementary material for: Evaluation of Methods for the Concentration and Extraction of Viruses from Sewage in the Context of Metagenomic Sequencing
Source: PLoS One. 2017 Jan 18;12(1):e0170199. doi: 10.1371/journal.pone.0170199 (PMC5242460; doi:10.1371/journal.pone.0170199)
Supplement: S4 Fig — Heatmap showing the abundance of all detected viral families, measured in reads per million, in each biological replica for the different method combinations as well as the controls. _S = sample, _C = Negative control. (PDF) [file pone.0170199.s004.pdf]

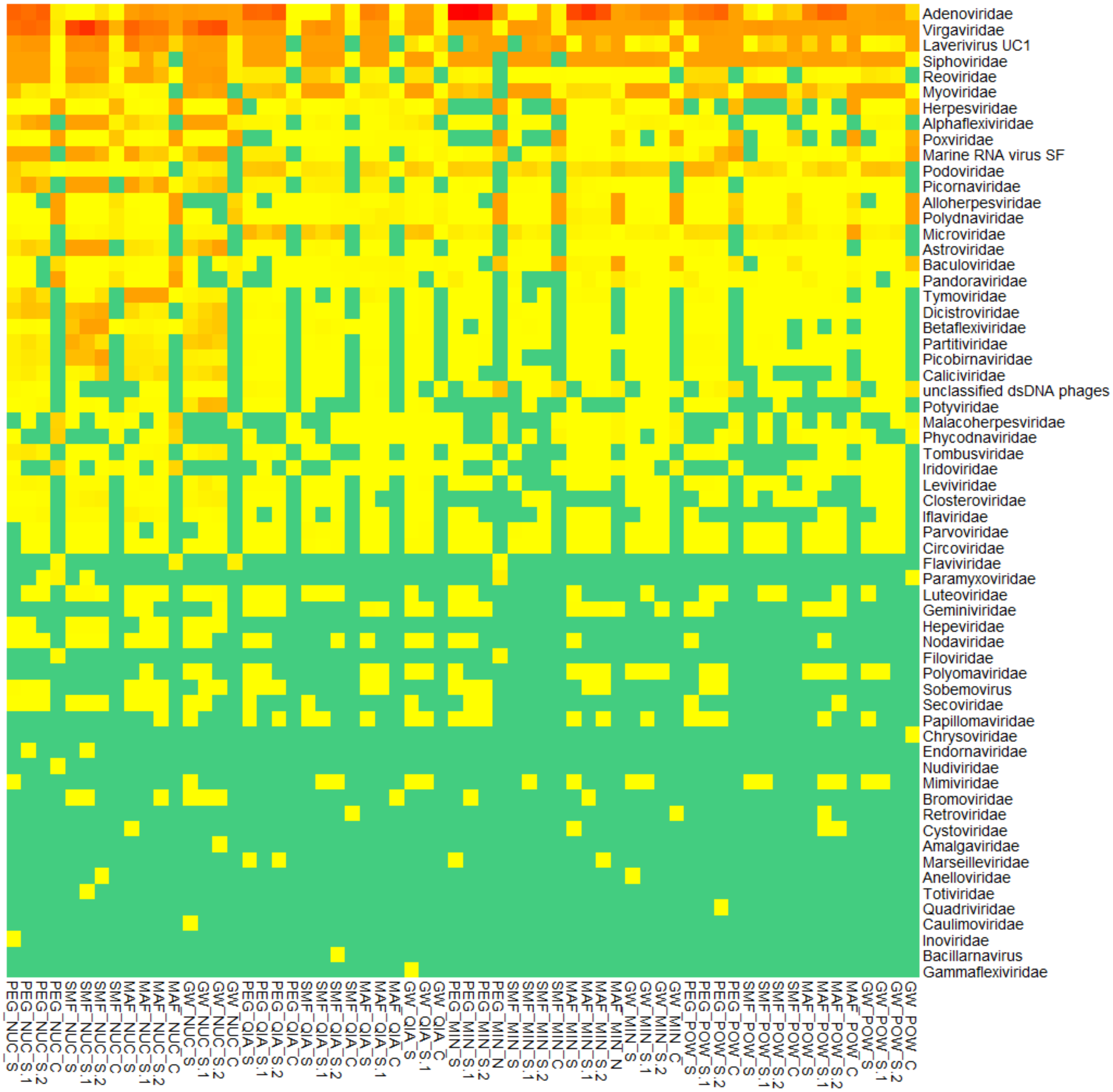

S4 Fig. **Abundance of all detected viral families.** Heatmap showing the abundance of all detected viral families, measured in reads per million, in each biological replica for the different method combinations as well as the controls. _S = sample, _C = Negative control
