# Supplementary material for: Evaluation of Methods for the Concentration and Extraction of Viruses from Sewage in the Context of Metagenomic Sequencing
Source: PLoS One. 2017 Jan 18;12(1):e0170199. doi: 10.1371/journal.pone.0170199 (PMC5242460; doi:10.1371/journal.pone.0170199)
Supplement: S1 Table — Number of raw reads, reads after quality assessment, and reads not mapping to PhiX, and thus usable for subsequent analysis. _S = sample, _C = Negative control. (PDF) [file pone.0170199.s005.pdf]

**S1 Table**. **Number of reads.** Number of raw reads, reads after quality assessment, and reads not mapping to PhiX, and thus usable for subsequent analysis. _S = sample, _C = Negative control

| Sample | Raw Reads | After Quality Assessment | notPhiX |
|---|---|---|---|
| **PEG_NUC_S** | 3923962 | 3762219 | 3705374 |
| **PEG_NUC_S** | 3754402 | 3578360 | 3532436 |
| **PEG_NUC_S** | 4271192 | 4095460 | 4014236 |
| **PEG_NUC_C** | 559652 | 456178 | 443970 |
| **SMF_NUC_S** | 4508374 | 4332741 | 4264728 |
| **SMF_NUC_S** | 4360562 | 4192762 | 4130628 |
| **SMF_NUC_S** | 4241578 | 4084436 | 4026938 |
| **SMF_NUC_** | 1101064 | 1003153 | 988386 |
| **MAF_NUC_S** | 4259050 | 4084200 | 4030550 |
| **MAF_NUC_S** | 3719448 | 3529342 | 3494230 |
| **MAF_NUC_S** | 3566548 | 3394287 | 3360646 |
| **MAF_NUC_C** | 157096 | 119941 | 116114 |
| **GW_NUC_S** | 3411102 | 3289449 | 3215330 |
| **GW_NUC_S** | 2828116 | 2728447 | 2658842 |
| **GW_NUC_S** | 3295268 | 3141210 | 3017800 |
| **GW_NUC_C** | 17392 | 14362 | 13636 |
| **PEG_QIA_S** | 3801098 | 3634483 | 3495492 |
| **PEG_QIA_S** | 2305672 | 2185372 | 2080556 |
| **PEG_QIA_S** | 4290068 | 4145091 | 4034670 |
| **PEG_QIA_C** | 4333452 | 4175469 | 4073050 |
| **SMF_QIA_S** | 4563326 | 4418363 | 4320250 |

| | | | |
|---|---|---|---|
| **SMF_QIA_S** | 2304182 | 2220290 | 2152844 |
| **SMF_QIA_S** | 3379888 | 3333397 | 3312860 |
| **SMF_QIA_C** | 3146584 | 3099536 | 3071026 |
| **MAF_QIA_S** | 1964426 | 1941789 | 1925920 |
| **MAF_QIA_S** | 1935286 | 1911652 | 1894644 |
| **MAF_QIA_C** | 2738054 | 2698372 | 2676234 |
| **GW_QIA_S** | 3721384 | 3626310 | 3605338 |
| **GW_QIA_S** | 2472348 | 2420355 | 2406568 |
| **GW_QIA_C** | 2300600 | 2217212 | 2200980 |
| **PEG_MIN_S** | 3698374 | 3633079 | 3600800 |
| **PEG_MIN_S** | 2738196 | 2697360 | 2668238 |
| **PEG_MIN_S** | 1397598 | 1364119 | 1344608 |
| **PEG_MIN_C** | 133788 | 123893 | 120862 |
| **SMF_MIN_S** | 2071278 | 2035964 | 2010478 |
| **SMF_MIN_S** | 1530636 | 1495490 | 1467210 |
| **SMF_MIN_S** | 1380108 | 1345321 | 1318090 |
| **SMF_MIN_C** | 67886 | 61172 | 58602 |
| **MAF_MIN_S** | 4847266 | 4740720 | 4647822 |
| **MAF_MIN_S** | 3504602 | 3437665 | 3380912 |
| **MAF_MIN_S** | 3932158 | 3851802 | 3781236 |
| **MAF_MIN_C** | 569434 | 545835 | 533460 |
| **GW_MIN_S** | 4373040 | 4276262 | 4213678 |
| **GW_MIN_S** | 3547238 | 3469203 | 3406014 |
| **GW_MIN_S** | 4630548 | 4519100 | 4442998 |
| **GW_MIN_C** | 753804 | 717635 | 705386 |

| | | | |
|---|---|---|---|
| **PEG_POW_S** | 5761750 | 5654298 | 5569710 |
| **PEG_POW_S** | 5576440 | 5478318 | 5414066 |
| **PEG_POW_S** | 2058156 | 2020902 | 2007878 |
| **PEG_POW_C** | 248950 | 231433 | 228512 |
| **SMF_POW_S** | 4255058 | 4185925 | 4148056 |
| **SMF_POW_S** | 3539780 | 3490092 | 3458870 |
| **SMF_POW_S** | 3580896 | 3532534 | 3497672 |
| **SMF_POW_** | 1449418 | 1404258 | 1393284 |
| **MAF_POW_S** | 5355902 | 5272249 | 5206222 |
| **MAF_POW_S** | 3573108 | 3459546 | 3395826 |
| **MAF_POW_S** | 3922566 | 3795625 | 3722032 |
| **MAF_POW_C** | 105730 | 85315 | 83474 |
| **GW_POW_S** | 3202862 | 3127023 | 3074676 |
| **GW_POW_S** | 4823400 | 4769483 | 4732332 |
| **GW_POW_S** | 3343808 | 3276568 | 3241548 |
| **GW_POW_C** | 111302 | 105263 | 103502 |
