# Supplementary material for: Evaluation of Methods for the Concentration and Extraction of Viruses from Sewage in the Context of Metagenomic Sequencing
Source: PLoS One. 2017 Jan 18;12(1):e0170199. doi: 10.1371/journal.pone.0170199 (PMC5242460; doi:10.1371/journal.pone.0170199)
Supplement: S2 Table — Reference sequence information can be obtained from the URL’s shown in ‘Download information’. (PDF) [file pone.0170199.s006.pdf]

S2 Table. **Overview of reference sequence databases and associated download information.**

Reference sequence information can be obtained from the URL's shown in 'Download information'.

Database name	Download information
**MetaHitAssembly**[2]	http://www.ncbi.nlm.nih.gov/nuccore/?term=PRJEB674 (PRJEB674 - PRJEB1046)
**Bacteria**[2]	ftp://ftp.ncbi.nih.gov/genomes/genbank/bacteria
**Human**	http://www.ncbi.nlm.nih.gov/assembly/GCF_000001405.25/#/def_asm_Primary_Assembly<br>Human genome ver. GRCh37.p13
**Invertebrates**	ftp://ftp.ncbi.nlm.nih.gov/ genomes/genbank/invertebrate
**Protozoa**	ftp://ftp.ncbi.nlm.nih.gov/ genomes/genbank/protozoa
**Virus**[1]	ftp://ftp.ncbi.nih.gov/genomes/virus
**Virus_NCBI**[1]	http://www.ncbi.nlm.nih.gov/genbank/
**Virus_Vipr**[1]	http://www.ncbi.nlm.nih.gov/pmc/articles/PMC3245011
**Plants**	ftp://ftp.ncbi.nlm.nih.gov/ genomes/genbank/plant
**Fungi**	ftp://ftp.ncbi.nlm.nih.gov/ genomes/genbank/fungi
**Parasites**	ftp://ftp.ncbi.nlm.nih.gov/genomes/genbank/parasites
**Vertebrates**	ftp://ftp.ncbi.nlm.nih.gov/genomes/genbank/vertebrates_mammals<br>ftp://ftp.ncbi.nlm.nih.gov/genomes/genbank/vertebrates_other

[1] All viral databases were combined into one. [2] All bacterial databases were combined into one.
