# Supplementary material for: Evaluation of Methods for the Concentration and Extraction of Viruses from Sewage in the Context of Metagenomic Sequencing
Source: PLoS One. 2017 Jan 18;12(1):e0170199. doi: 10.1371/journal.pone.0170199 (PMC5242460; doi:10.1371/journal.pone.0170199)
Supplement: S3 Table — Inhibition of the 16 combinations of concentration and extraction methods. Inhibition was measured using qPCR of undiluted (1:1) and tenfold diluted (1:10) DNA/RNA extracts. The values in the tables represents Δct between the undiluted and 10 fold diluted samples. A Δct = -3.3 represent a perfect 10 fold dilution. Samples marked in red represents undiluted extracts that could not be quantifiable, these samples are regarded as the most inhibited. (PDF) [file pone.0170199.s007.pdf]

S3 Table. **qPCR inhibition of MNV and HAdV.** Inhibition of the 16 combinations of concentration and extraction methods. Inhibition was measured using qPCR of undiluted (1:1) and tenfold diluted (1:10) DNA/RNA extracts. The values in the tables represents Δct between the undiluted and 10 fold diluted samples. A Δct = -3.3 represent a perfect 10 fold dilution. Samples marked in red represents undiluted extracts that could not be quantifiable, these samples are regarded as the most inhibited.

Method	MNV	HAdV
PEG_NUC	-1.80	1.07
SMF_NUC		
MAF_NUC	-2.38	-3.65
GW_NUC	-0.58	-2.08
PEG_QIA	-1.78	-2.78
SMF_QIA		
MAF_QIA	0.65	
GW_QIA	-0.45	
PEG_MIN	-3.04	-2.34
SMF_MIN	0.04	-3.51
MAF_MIN	-3.14	-2.17
GW_MIN	-2.25	-2.69
PEG_POW	-0.06	-1.79
SMF_POW		
MAF_POW	-0.42	3.07
GW_POW		0.52
