# Supplementary material for: Evaluation of Methods for the Concentration and Extraction of Viruses from Sewage in the Context of Metagenomic Sequencing
Source: PLoS One. 2017 Jan 18;12(1):e0170199. doi: 10.1371/journal.pone.0170199 (PMC5242460; doi:10.1371/journal.pone.0170199)
Supplement: S4 Table — (PDF) [file pone.0170199.s008.pdf]

S4 Table. **Specifications of the four concentration methods applied in this study.**

	PEG 8000 Precipitation (PEG)	Glass Wool Filtration (GW)	Monolithic Affinity Filtration (MAF)	Skimmed Milk Flocculation (SMF)
**Primary Concentration Technology**	Precipitation	Filtration	Filtration	Precipitation
**Secondary Concentration Technology**	none	PEG 8000 precipitation	100 kDa ultracentrifugation filters	Ultracentrifugation
**Maximum input volume (ml sewage)**	500	4000	1000	10000
**Required machinery**	Centrifuge	Peristaltic pump	Peristaltic pump	Centrifuge Ultracentrifuge
**Price per sample (Euro)**	~20	~7	~3	~10
**Length of procedure (per sample h)**	~24	~ 24	~ 1.5	~24
**Minimum elution volume (ml)**	1	1	3	2
**Possibility of automatization**	no	no	(yes)*	no

. *In development
