# Supplementary material for: Evaluation of Methods for the Concentration and Extraction of Viruses from Sewage in the Context of Metagenomic Sequencing
Source: PLoS One. 2017 Jan 18;12(1):e0170199. doi: 10.1371/journal.pone.0170199 (PMC5242460; doi:10.1371/journal.pone.0170199)
Supplement: S5 Table — (PDF) [file pone.0170199.s009.pdf]

S5 Table. **Properties of the four nucleic acid extraction kits, QIA, NUC, MIN and, POW applied in this study.**

	QIAamp Viral RNA Mini Kit (QIA)	Nucleospin RNA XS (NUC)	NucliSENS® miniMAG® (MIN)	PowerViral® Environmental RNA/DNA Isolation Kit (POW)
**Technology**	Spin column	Spin column	Magnetic silica beads	Spin column
**Maximum input volume (µl)**	560	NA	1000	200
**DNase step included**	No	Yes	No	No
**Price for 50 samples (Euro)**	231	317	304	395
**Time for 24 samples (h)**	~ 3 hours	~ 3 hours	~ 3 hours	~ 3 hours
**Minimum elution volume (µl)**	30	5	25	50
**Possibility of automatization**	yes	no	yes	no
**Producer**	Qiagen	Macherey-Nagel	BioMérieux	MO BIO
